# CD24 Expression Identifies Teratogen-Sensitive Fetal Neural Stem Cell Subpopulations: Evidence from Developmental Ethanol Exposure and Orthotopic Cell Transfer Models

**DOI:** 10.1371/journal.pone.0069560

**Published:** 2013-07-22

**Authors:** Joseph D. Tingling, Shameena Bake, Rhonda Holgate, Jeremy Rawlings, Phillips P. Nagsuk, Jayashree Chandrasekharan, Sarah L. Schneider, Rajesh C. Miranda

**Affiliations:** 1 Department of Neuroscience & Experimental Therapeutics, Texas A&M Health Science Center, Bryan, Texas, United States of America; 2 Women’s Health in Neuroscience Program, Texas A&M Health Science Center, Bryan, Texas, United States of America; National Institutes of Health, United States of America

## Abstract

**Background:**

Ethanol is a potent teratogen. Its adverse neural effects are partly mediated by disrupting fetal neurogenesis. The teratogenic process is poorly understood, and vulnerable neurogenic stages have not been identified. Identifying these is a prerequisite for therapeutic interventions to mitigate effects of teratogen exposures.

**Methods:**

We used flow cytometry and qRT-PCR to screen fetal mouse-derived neurosphere cultures for ethanol-sensitive neural stem cell (NSC) subpopulations, to study NSC renewal and differentiation. The identity of vulnerable NSC populations was validated in vivo, using a maternal ethanol exposure model. Finally, the effect of ethanol exposure on the ability of vulnerable NSC subpopulations to integrate into the fetal neurogenic environment was assessed following ultrasound guided, adoptive transfer.

**Results:**

Ethanol decreased NSC mRNAs for c-kit, Musashi-1and GFAP. The CD24^+^ NSC population, specifically the CD24^+^CD15^+^ double-positive subpopulation, was selectively decreased by ethanol. Maternal ethanol exposure also resulted in decreased fetal forebrain CD24 expression. Ethanol pre-exposed CD24^+^ cells exhibited increased proliferation, and deficits in cell-autonomous and cue-directed neuronal differentiation, and following orthotopic transplantation into naïve fetuses, were unable to integrate into neurogenic niches. CD24^depleted^ cells retained neurosphere regeneration capacity, but following ethanol exposure, generated increased numbers of CD24^+^ cells relative to controls.

**Conclusions:**

Neuronal lineage committed CD24^+^ cells exhibit specific vulnerability, and ethanol exposure persistently impairs this population’s cell-autonomous differentiation capacity. CD24^+^ cells may additionally serve as quorum sensors within neurogenic niches; their loss, leading to compensatory NSC activation, perhaps depleting renewal capacity. These data collectively advance a mechanistic hypothesis for teratogenesis leading to microencephaly.

## Introduction

Early developmental experiences are increasingly recognized to be an important causative factor in adult neuropsychiatric diseases [1]. Fetal exposure to ethanol is an important example of an early developmental experience that results in long term brain and neurobehavioral deficits [2], [3], that are collectively termed the Fetal Alcohol Spectrum Disorder (FASD). Despite being identified as teratogenic for more than four decades [4], [5], *in utero* ethanol exposure continues to be a leading non-genetic cause of mental retardation. The incidence of Fetal Alcohol Syndrome, which represents the severe end of the FASD continuum, has persistently remained at 0.2%–0.7%, while the incidence of FASD is estimated to be between 2%–5% of the U.S. population [6]. An important question is, “why are developing fetal organs like the brain are so sensitive to teratogenic agents like ethanol?” Answers to this question are a prerequisite for the development of successful interventional programs to mitigate the effects of teratogens.

A majority of women who consume alcohol during pregnancy, do so during the first and second trimester, and usage declines dramatically in the third trimester [7]. The end of the first trimester and the beginning of the second trimester constitute an important developmental period where neural stem cells (NSCs) within fetal ventricular zones generate most of the neurons of the adult brain (for review see [8]). Consequently, maternal alcohol consumption patterns are statistically likely to bracket this important period of neurogenesis in the developing fetal brain. Several laboratories have shown that ethanol exposure near the end of the first [9] and second trimester-equivalent period [10]–[16] can lead to persistent changes in brain structure. These data suggested, but did not specifically show that cells within the fetal neuroepithelium were directly vulnerable to ethanol. We [17]–[19], and others [20]–[23] specifically identified fetal neural epithelial cells as a vulnerable target of ethanol, in that ethanol exposure resulted in both immediate and persistent alterations in neuroepithelial renewal and differentiation, importantly, without inducing cell death [17], [23], [24]. This indicates that ethanol does not behave as a toxin in the fetal neuroepithelium, but as a true teratogen. The fetal neuroepithelium is a complex neurogenic niche. During the second trimester equivalent period, NSCs undergo renewal, or alternatively, following activation, generate daughter progenitors in a series of steps, from transit amplifying precursors, to neuronal lineage committed precursors. Lineage committed precursors migrate away from the ventricular zone (VZ) to intermediate germinal zones like the sub-ventricular zone (SVZ) before finally differentiating into neurons (reviewed in [25]). We specifically found that ethanol stimulated neuroepithelial cell proliferation while decreasing NSC characteristics and promoting aberrant differentiation. From these data, we hypothesized that ethanol depleted fetal NSCs, not by inducing cell death, but by promoting their transformation to transit amplifying cells and consequently, premature differentiation.

It is important to identify specific stages of NSC maturation that are selectively vulnerable to teratogens like ethanol. Such evidence would serve to focus future research on reprogramming targeted NSC maturation stages to mitigate the severity of fetal brain damage. We followed an increasingly utilized approach to identifying and categorizing neuroepithelial cells by their complement of cell surface immunologic markers [26]–[28]. Collectively, these markers appear to constitute a molecular code for the identity of neuroepithelial cells at different maturation stages. We identified CD24^+^ cells, and more specifically, the CD24^+^CD15^+^ double-positive population as a specific target of ethanol. In both *in vitro* and in orthotopic adoptive-transfer experiments, we found that ethanol exposure renders the CD24^+^ subpopulation insensitive to environmental manipulation suggesting that ethanol exposure results in cell-autonomous re-programming of the CD24^+^ population, and that the underlying mechanisms for programming CD24^+^ NSCs perhaps constitute a specific locus for teratogenesis.

## Methods

### Ethics Statement

Timed-pregnant C57Bl/6 mice (Harlan, Texas) were housed in an AAALAC-approved facility at TAMHSC. All protocols were conducted with the approval of the Institutional Animal Care and Use Committee at TAMU (Approval number AUP2010-197).

### Neurosphere Culture

Fetal cerebral cortical neuroepithelial cells were obtained from gestational day (GD) 12.5 (GD0 was defined as the day of mating when the dam was sperm plug-positive) mouse dorsal pallium, corresponding to the structural precursor of the mouse iso-cortex, according to previously published protocols [17]. Neuroepithelial cells collected from an entire litter, were dispersed in a defined culture medium DMEM F12 (Invitrogen, #11039-047), 20 ng/ml Basic Fibroblast Growth Factor (bFGF, BD Biosciences, #354060), 20 ng/ml human recombinant Epidermal Growth factor (EGF, Invitrogen #47743-568), 0.15 ng/ml recombinant human Leukemia Inhibitory Factor (LIF, Alomone, #L-200), ITS (Insulin, Transferrin, Selenium)-X supplement (Invitrogen, #51500-056), 0.85 units/ml Heparin (Sigma-Aldrich, #H-4784), 20 nM progesterone (Sigma-Aldrich, #P7556) and cultured as non-adherent neurosphere aggregates. Independent replicates for the reported experiments were from neurospheres established from a total of 12 different pregnancies.

Neurosphere cultures were randomly assigned to groups; control and ethanol-exposed (320 mg/dl (70 mM) or 120 mg/dl (26 mM) ethanol, prepared from 95% ethanol Sigma-Aldrich 190 proof molecular biology grade (E7148)), according to previous protocols [19]. The dose of ethanol was selected to be within the range attained in chronic alcoholics [29], and represents a dose that is known to influence the maturation of NSCs without causing apoptosis [23], [24]. The final ethanol concentration in the culture media was determined by gas chromatography, from samples of the media collected at the beginning of the treatment at day 0, at day 3 for re-feeding and at day 5 before collection. To assess the differentiation of ethanol pre-exposed NSC subpopulations, cells were cultured on a laminin-coated substrate for three days, in the absence of EGF and LIF (mitogen-withdrawal differentiation paradigm) or in the presence of retinoic acid (1 µM, cue-directed differentiation paradigm). In each experiment, control cells were paired with ethanol-treated cells and cultured under the same conditions and for the same length of time as ethanol-exposed cells.

The intracellular fluorescent dye, carboxyfluorescein succinimidyl ester (CFSE, Life Technologies) was used to label cells at a concentration of 10 µM for cell proliferation studies in which cells were labeled with dye after five-day ethanol treatment withdrawal and analyzed over three days. A decrease in cell fluorescence over time is an index of proliferative activity, as successive cell division cycles result in dye dilution [30]. As a second measure of cell proliferation, DNA synthesis was measured using the Click-iT® EdU cell proliferation assay (Life Technologies, #C-10425) according to the manufacturers instructions. Briefly, 24 hours following pre-treatment with ethanol or control medium, cells were exposed to 10 µM EdU (5-ethynyl-2′-deoxyuridine) for 30 minutes, for incorporation of EdU into DNA of cells in S-phase. Incorporated EdU was detected by covalently binding Alexa Fluor® 488-azide, followed by flow cytometry.

### Maternal Ethanol Exposure Paradigm

Ethanol (3 g/kg b. wt.) or an equal volume of water was administered to timed-pregnant C57Bl/6 female mice, twice daily by intragastric gavage for 3 days, from GD12.5 through GD14.5, to bracket the first half of the second trimester-equivalent period of neurogenesis. We previously showed that this level of exposure results in an average maternal peak blood alcohol concentration of 117 mg/dl [31], and is readily attainable in human populations. At 30 minutes following the final treatment, animals were deeply anesthetized with xylazine and ketamine (0.l ml/g b. wt. of a mixture of 1% xylazine and 0.25% ketamine). Fetuses were dissected out of uterine horns, fixed in 4% paraformaldehyde overnight, transferred to PBS, and stored at 4°C. Fetuses (one from each pregnant dam for a sample size of 4 in each treatment condition) were embedded in paraffin blocks (two control and two ethanol exposed fetuses per block), sectioned in sagittal orientation at 7 µm thickness, and mounted on charged slides (Thermo Scientific, ColorFrost-plus).

### Real-Time PCR

Total mRNA was purified from neurosphere cultures using the mirVana miRNA Isolation Kit (Life Technologies, #AM1561) using the total RNA isolation protocol. Quantification of mRNA was done using a ThermoScientific NanoDrop 2000 spectrophotometer. cDNA synthesis at 1 µg concentration was performed using qScript cDNA Supermix (Quanta Biosciences, #95048-500). Forward and reverse primers were designed based on Harvard primer bank and verified using UCSC Genome BLAT browser. Selected primers (**Table 1**) were then optimized by a standard curve using serial dilutions of the template to determine the amplification efficiency of the PCR reaction. The amplification of a single transcript was verified by thermal stability analysis. For real-time PCR, cDNA, forward and reverse primers, and Quanta Biosciences PerfeCta SYBR Green SuperMix for iQ (#95053-500) were combined and analyzed with the iCycler MyiQ system from BioRad.

**Table 1:** List of primers for qRT-PCR analysis

Gene	Forward primer	Reverse primer
**Map-2**	5'- CTGGACATCAGCCTCACTCA-3'	5'-AATAGGTGCCCTGTGACCTG-3'
**Gfap**	5'-GCTTCCTGGAACAGCAAAAC-3'	5'-ATCTTGGAGCTTCTGCCTCA-3'
**c-Kit**	5'-GCCACGTCTCAGCCATCTG-3'	5′-GTCGCCAGCTTCAACTATTAACT-3'
**Sox-3**	5'-CGTAACTGTCGGGGTTTTGT-3'	5'-AACCTAGGAATCCGGGAAGA-3'
**Sox-9**	5′-ATAAGTTCCCCGGTGCATC-3'	5'-TACTGGTCTGCCAGCTTCCT-3'
**Musashi-1**	5'-GCTACTGCCTGTCCCTCAAC-3'	5'-GGGTAGGGCAACTGGCTAAT-3'
**Nestin**	5'-CCAGAGCTGGACTGGAACTC-3'	5'-ACCTGCCTCTTTTGGTTCGT-3'
**18s**	5'-ATGGCCGTTCTTAGTTGGTG-3'	5'- CGCTGAGCCAGTCAGTGTAG-3'

### Flow Cytometry

A Guava EasyCyte Plus Bench-Top Flow Cytometer (Millipore) was used to determine expression of stem cell markers. Antibodies were obtained from Abcam for CD24 PE (phycoerythrin)-conjugated (ab25646), CD90/Thy1.1 PE-conjugated (ab24904), CD15/SSEA-1 (ab16285), CD34 PE-conjugated (ab23830), CD43 FITC-conjugated (ab21853), CD105 (ab21222). All antibodies were used at a 1∶100 concentration. We selected several cell surface markers that are known to identify activated NSCs, transit amplifying stem cells and neuroblasts. CD15/SSEA-1 is a marker of pluripotent embryonic stem cells [27], [32]–[36] and was chosen to identify NSCs that retain pluripotency. CD34 is a glycophosphoprotein that is expressed in a variety of stem cell populations including NSCs and HSCs [37]–[39] and also identifies an early NSC phenotype. CD49f and CD90 (Thy-1.1) mark transit-amplifying cells [40], [41] that arise from activated NSCs, though CD90 continues to be expressed in sub-populations of mature neurons [42]. CD24 has been identified as a marker of transit amplifying cells as well as neuronal lineage commitment in the mouse [26], [27], [43]. The loss of co-localization of CD15 with CD24 has been shown to define a shift between NSCs/transit-amplifying cells and neuronal lineage committed neuroblasts [27]. CD105 and CD43, in contrast, identify endothelial and hematopoietic lineages [44], [45], and the expression of these markers was undetectable (data not shown), indicating that there was not significant cross contamination of neurosphere-cultured cells. Flow cytometry studies quantified immunolabeling from a sample of 10,000 cells, and the data obtained from triplicate technical replicates were averaged prior to statistical analysis. Isotype-specific antibody controls were used to threshold background antibody labeling and forward scatter (FSC) parameter was used to gate cell size.

### Apoptosis Assay

Apoptosis was detected by a Caspase activity assay (Abcam, ab112130). Briefly, control, ethanol-pretreated, and as a positive control, Staurosporine (2 µM for two hours)-treated cells were dispersed into a single-cell suspension and counted. An equal number of cells (8.5×10^5^ cells/ml) from each condition were exposed to the cell-permeable reagent, TF2-VAD-FMK, which irreversibly binds activated Caspases, for four hours. Cells were then washed and activated Caspase activity determined on a fluorescence plate reader (Tecan Infinite M200), at an excitation frequency of 490 nm and emission frequency of 525 nm. An aliquot of cells were also counter-stained with DAPI to label nuclei, and placed on a slide for visualization by fluorescence microscopy.

### Immunofluorescence Analysis

Differentiating NSCs were cultured on plastic culture coverslips (Cat#174977; Thermanox) in 6-well plates for periods up to 36 hours, then fixed in ice-cold methanol for 30 minutes and rinsed 3× with PBS. Immunofluorescence analysis was conducted with primary antibodies to β-Catenin (#610153, 1∶300 µL; BD Biosciences), visualized with Alexa Fluor-488 (A11001; Invitrogen) conjugated goat anti-mouse secondary antibody, or primary antibodies to MAP2 a&b (Ab5622, 1∶300 µL; Millipore) and Nestin (Ab7659; Abcam), visualized with Alexa Fluor-594 (A11032; Invitrogen), Alexa Fluor-488 (A11001; Invitrogen), conjugated goat anti-rabbit secondary antibodies, respectively. Nuclei were visualized by counter-staining with DAPI (H-1200; Vector), and antibody binding was visualized by epifluorescence imaging on an Olympus BX60 microscope. Paraffin embedded tissue sections of fetal brain (one from each pregnant dam, for a total of four control and four ethanol exposed fetuses) were deparaffinized and assessed for the expression of CD24 by direct immunofluorescence using the same antibody (PE-conjugated anti-CD24 (ab25646, Abcam)) that was used for flow cytometric analysis to provide for equivalent assessment of staining with the two protocols.

### Immuno-magnetic Cell Selection and Depletion

CD24^+^ and CD24^depleted^ cell populations were isolated for further study, using immunomagnetic-selection and –depletion protocols (Stem Cell Technologies EasySep PE Selection Kit (#18551)) with a Stem Cell Technologies RoboSep Automated Cell Isolation system. Briefly, cells were incubated with CD24-PE conjugated primary antibody (at a 1∶10 dilution) for 15 minutes, followed by addition of EasySep PE Selection Cocktail (Stem Cell Technologies) at 100 µl/ml, and incubated for 15 minutes. A 50 µl/ml solution of EasySep magnetic particle solution was then added and incubated for 5 minutes. Following 5 washes in 1× PBS, both positive selected (CD24^+^) and depleted (CD24^depleted^) cells were collected and concentrated. After quantification, between 10^4^–10^5^ cells were resuspended in a final volume of 100 µl PBS for further analyses. For *in utero* adoptive transfer experiments, isolated CD24^+^ cells were tagged with a fluorescent dye (Vybrant DiD (Life Technologies, V-22887) or Vybrant DiO (Life Technologies, V-22886). A solution, containing equal numbers of control and ethanol treated CD24+ isolated cells, was aspirated into a quartz microcapillary pipette in preparation for ultrasound guided intrauterine injections.

### Ultrasound Guided Trans-Uterine Neural Stem Cell Microinjection

Anesthesia was induced in timed-pregnant mice with an initial mixture of 0.4% medical grade oxygen and 4% Isoflurane (Butler, #029495), and maintained with a 0.4% Oxygen and a 2% Isoflurane mixture during the surgery. The surgical procedure was conducted within a sterile HEPA-filtered field (LabConco PuriCare Open Access Station) according to approved protocol. Maternal electrocardiogram and core body temperature were monitored using an Indus Instruments, Temperature and ECG Monitoring system (#THM100). Core body temperature was maintained at 33–35°C and maternal heart rate was monitored with adjustments to the level of anesthesia made to maintain a constant heart rate of ∼450 beats/minute. The abdomen was shaved and depilated (using NAIR®) and the planned surgical incision area was pre-sterilized with ethanol (80% v/v) followed by Betadine®. Brief ultrasound imaging was conducted to determine the number and position of embryos present in each pregnant mouse. A 15 mm-long vertical incision was made, 1 mm lateral to the midline and medial to superficial epigastric artery, and the outer skin retracted. A 10–12 mm incision was then made through the peritoneal wall. One of the uterine horns was selected for microinjection and gently exteriorized using sterile cotton-tipped applicators dipped in sterile saline. Fetuses were counted from the fetus that was in the most proximal position to the ovary. The uterus was gently pulled through the base of a sterile 15 mm petri dish through a circular hole covered with a perforated sterile film. Ultrasound gel (Ecogel, CA) was pre-warmed and applied to the uterus. A VisualSonics Vevo 2100 high-frequency, high-resolution digital imaging platform, coupled to a MS550D Microscan™ transducer with a center frequency of 40 MHz, an Integrated Rail System III with Microinjector, and animal handling and physiology monitoring platform (Visualsonics, Toronto, Canada), was used to visualize GD13 fetuses after surgical dissection of the abdominal wall allowing for trans-uterus image-guided microinjection into two fetuses. The trans-abdominal incision was sutured using a silk suture thread, Orajel™ and Neosporin™ were then applied to the open incision and the skin was closed using mice wound closure clips. Mice were monitored and observed for full recovery. Pregnant mice were euthanized at 48 and 72 hours after microinjections by anesthesia with ketamine (0.09 mg/gram)/xylazine (0.106 mg/gram) *via* an intraperitoneal injection. The gravid uterus was excised and each fetus was removed from its embryonic sac, fixed in a 4% paraformaldehyde solution for 24 hours, and subsequently cryo-protected with a 30% sucrose solution. Each fetus was then embedded using NEG50 Frozen Section Medium (Richard-Allan Scientific, #6502), cryo-sectioned at 20 µm, and imaged using an Olympus BX60 microscope. The number and location of each labeled cell in every section from a whole mouse embryo brain was counted in a blinded manner.

### Statistical Analysis

Data were analyzed by multivariate or univariate Analysis of Variance (MANOVA or ANOVA), followed by the Fisher’s least significant difference test (SPSS v20, IBM). Pair-wise comparisons were assessed using a t-test. Sample sizes were 4–5 independent replicates per treatment condition. Data were expressed as mean±standard error of the mean (SEM).

## Results

### Ethanol Decreases Expression of mRNAs that Support NSC Identity

**Figure 1:**
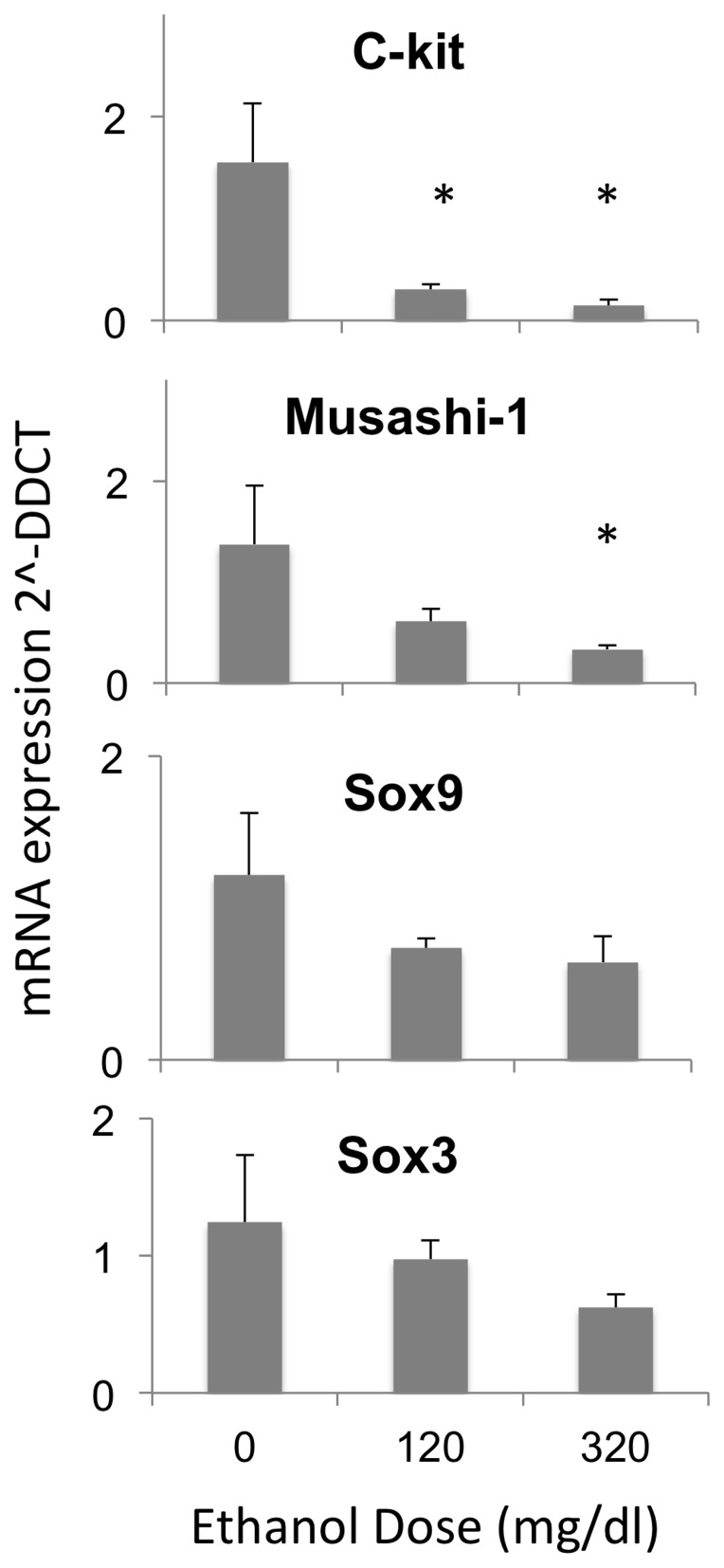
Ethanol effects on stem cell mRNAs. QRT-PCR analysis of mRNA expression of stem cell markers, expressed as fold-change relative to the mean of control, in control and ethanol treated mouse neurosphere cultures showing a general ethanol-related decrease in stem cell mRNAs and significant decline in c-Kit and Musashi-1 mRNA. Asterisks indicate statistically significant differences relative to controls.

**Figure 2:**
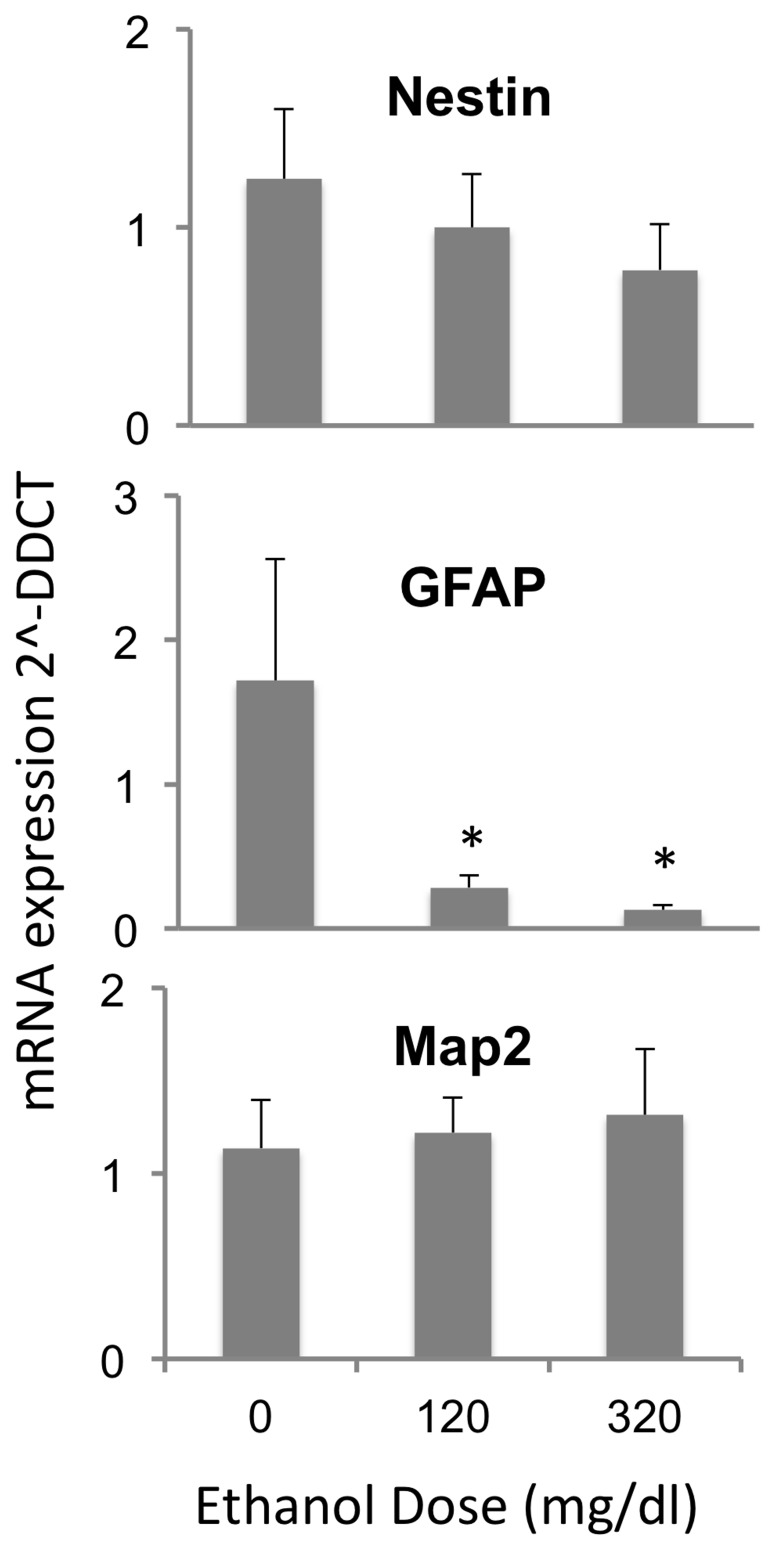
Ethanol effects on mRNA transcripts for cytoskeletal proteins. QRT-PCR analysis of mRNA expression for lineage-specific intermediate filament and microtubule-associated proteins, showing a statistically significant decrease in the expression of GFAP mRNA with ethanol exposure. Asterisks indicate statistically significant differences relative to controls.

We screened GD12.5 fetal mouse-derived neurosphere cultures for the expression of mRNA transcripts for transcription factors (Sox9 and Sox3, [46], [47]), RNA binding proteins (Musashi-1, [48]) and signaling molecules (c-kit, [49]) that are important for induction of neural identity in stem cells, and the maintenance and renewal of NSCs. Ethanol exposure resulted in a dose-related suppression of mRNA transcripts, for c-kit and Musashi-1 (F_(2,12)_ = 7.58, p<0.007, and F_(2,12)_ = 4.23, p<0.041 respectively, **Figure 1**). Both low and high ethanol doses significantly reduced c-kit mRNA (post-hoc, pair-wise comparisons, p<0.042 and p<0.002 respectively), and the high ethanol dose significantly reduced Musashi-1 mRNA (p<0.013). Ethanol did not significantly alter the expression of Sox9 and Sox3 (**Figure 1**). Next, we screened cultures for the expression of mRNA transcripts for intermediate filament and microtubule-associated proteins, which are important components of the identity of NSCs and their more mature daughter cells (**Figure 2**). Ethanol exposure led to a dose-related decrease in the expression of mRNA transcripts for GFAP (F_(2,12)_ = 8.31, p<0.005). Both low and high ethanol doses resulted in significantly decreased GFAP expression relative to controls (p<0.02 and 0.002 respectively). Transcripts for intermediate filaments broadly identifying NSC populations (nestin) and early neuronal identity (Map2) were not significantly altered following ethanol exposure. Nestin-positive NSCs include both GFAP-positive and GFAP-negative sub-populations [50], comprising early and late progenitor populations respectively [51]. Therefore, evidence for selective suppression of GFAP but not Nestin mRNA suggests that ethanol targets the early sub-population of NSCs. Moreover, since GFAP-positive NSCs, are capable of self-renewal [52], and since ethanol also decreased mRNA transcripts for NSC factors like c-kit and Musashi-1, these data suggested that ethanol leads to a partial loss of NSC renewal characteristics.

### Ethanol Exposure Prevents Subsequent Differentiation of NSCs

**Figure 3:**
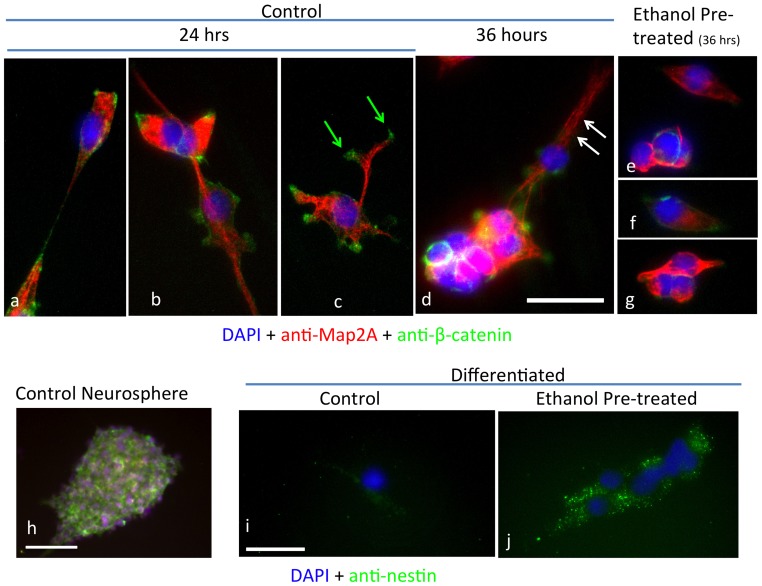
Ethanol effects on neural differentiation. Photomicrographs of immunofluorescence analysis for markers of differentiation in control and ethanol exposed cultures, counterstained with DAPI (blue fluorescence) to visualize nuclei. (**a–g**) Neurosphere cultures were treated with control medium (**a–d**) or 320 mg/dl ethanol (**e–g**) for 5 days, then differentiated in the absence of ethanol, on a laminin-coated substrate with the mitogen withdrawal paradigm (see methods). Cells cultured under control conditions express neuritis that fasciculate to form bundles (white arrows) and express Map2a/b and β-catenin (**d**, green arrows) immunofluorescence indicative of dendritogenesis. Cells obtained from ethanol pre-exposed neurosphere cultures express Map2a/b immunofluorescence but do not exhibit β-catenin immunofluorescence or evidence for dendritogenesis. (**h**) Sample neurosphere expressing nestin immunofluorescence (green) counterstained with DAPI. (**i–j**) Following exposure to control (**i**) or ethanol-containing medium (**j**) and differentiation induced by mitogen withdrawal, differentiating control cultures exhibit near undetectable levels of nestin immunofluorescence (green), while differentiating cells pre-exposed to ethanol continue to express nestin immunofluorescent puncta, suggesting the presence of de-polymerized nestin. Scale bars, **a–g**, 50 µm; **h**, 50 um; **i–j**, 50 µm.

We next asked if exposing NSCs to ethanol resulted in persistent defects in differentiation. We treated neurosphere cultures with ethanol or control medium for five days, and then exposed NSCs to a differentiation paradigm consisting of withdrawal of EGF and LIF and culturing cells on an extracellular matrix of laminin [19]. As predicted by our previous experiments [19], the mitogen-withdrawal differentiation paradigm results in morphological transformation of NSCs (**Figure 3a–d**). Control NSCs develop Map2-immunopositive processes terminating in β-catenin-positive lamellipodia (e.g., **Figure 3c**, arrows), showing the emergence of dendrite-like processes and neuronal lineage commitment [53]. Over a 36-hour period, increasing neurite outgrowth, migration and fasciculation of neurites was observed (**Figure 3d** **, arrows**), with a simultaneous loss of nestin immunoreactivity (compared to the presence of nestin in undifferentiated neurospheres, **Figure 3h** *vs*. **Figure 3i**). In contrast, differentiating NSCs pre-exposed to ethanol, exhibited shortened processes that lacked β-catenin-immunopositive lamellipodia (**Figure 3** **e–g**). Moreover, at 36 hours following differentiation, ethanol-pretreated cells remained immunopositive for nestin, though the staining was localized to discrete cytoplasmic puncta (**Figure 3j**) rather than to fibrils, suggesting the presence of depolymerized, residual nestin. Therefore, ethanol exposure resulted in decreased capacity for subsequent differentiation.

### Expression of Markers for NSC Identity and Lineage Commitment

**Figure 4:**
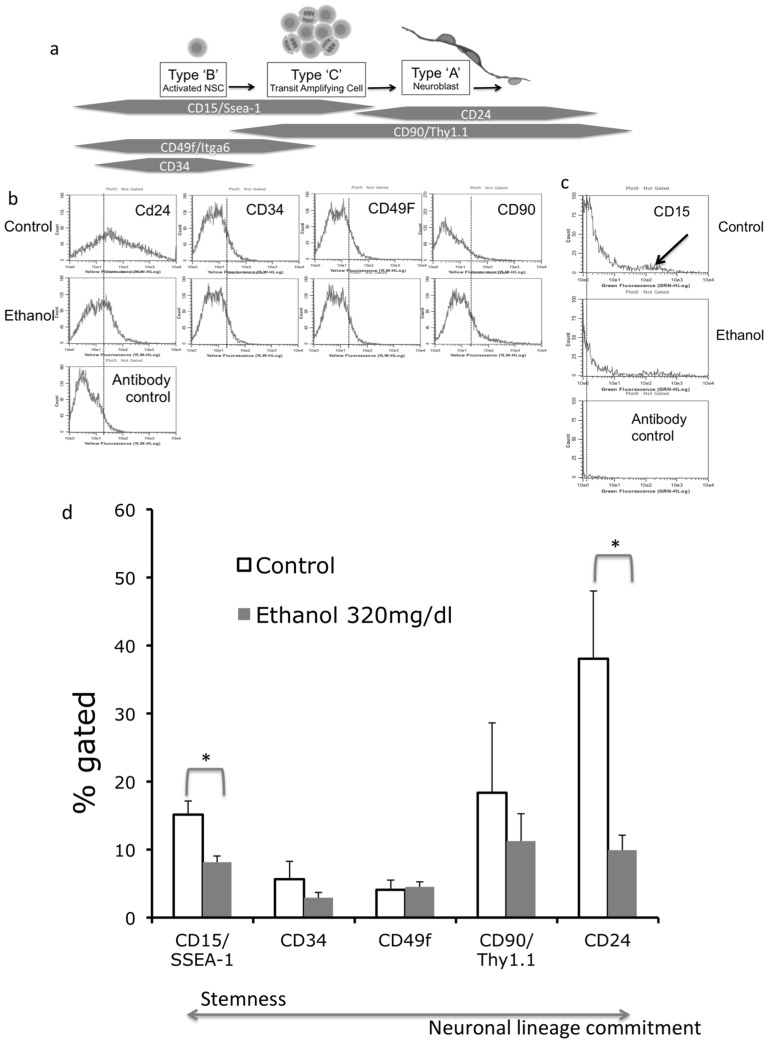
Expression and regulation of cell-surface markers. (a) Schematic diagram showing the stages of the progression of neural stem cells and their relation to the cell surface molecular map used in flow cytometric analysis. (**b–c**) Sample flow cytometric histograms for stem CD24, CD34, CD49f and CD90 (**b**), and CD15 (**c**, arrow identifies the CD15^High^ population), in control and ethanol-treated (320 mg/dl) cultures. ‘X’ axis indicates fluorescence intensity while ‘Y’ axis indicates cell number. The histogram in the bottom panel in ‘**b**’ and ‘**c**’ indicates background immunofluorescence visualized by staining with an isotype-specific antibody, used to gate specific labeling (vertical dashed lines). (**d**) Quantification of flow cytometric data shows that ethanol significantly decreased the numbers of cells specifically labeled with CD24 or CD15. Asterisks indicate statistically significant differences relative to controls.

In the next series of experiments, we screened for the expression of a variety of cell surface markers that are collectively known to span the transition from pluripotency, through transit amplification, to neuronal identity (**Figure 4a**) to identify specific ethanol susceptible NSC populations. Because of technical limitations with scaling-up cultures within a limited range of passage numbers to limit senescence-induced phenotypic variability, subsequent experiments utilized only the high dose of ethanol (320 mg/dl), where we observed the greatest decreases in mRNA expression.

Flow cytometric analyses showed that mouse neuroepithelial cells were heterogeneous with respect to their expression of a variety of cell surface markers; CD34 (5.6±2.6%), CD49f (4.1±1.4%), CD15 (15.2±2%), CD90 (18.4±10.3%) and CD24 (38.1±10%) (**Figure 4** **b,c**). However, specific immuno-labeling was not detected for markers of hematopoietic cells or endothelial cells (CD 43 and CD105 respectively, data not shown) suggesting that cultured neuroepithelial cells were uncontaminated with other proliferating cell populations. Interestingly, immuno-labeling for CD15 revealed at least two separate cell populations, a minor CD15^High^ population (**Figure 4c**, arrow), and a dominant population of cells that expressed more moderate levels of CD15 (CD15^Moderate^). In contrast, CD24 immuno-labeling revealed a broad peak of labeled cells, suggesting that this maker identifies a heterogeneous group of cells that express variable levels of cell surface CD24 expression.

### Ethanol Exposure Specifically Reduces CD15^+^ and CD24^+^ Populations

**Figure 5:**
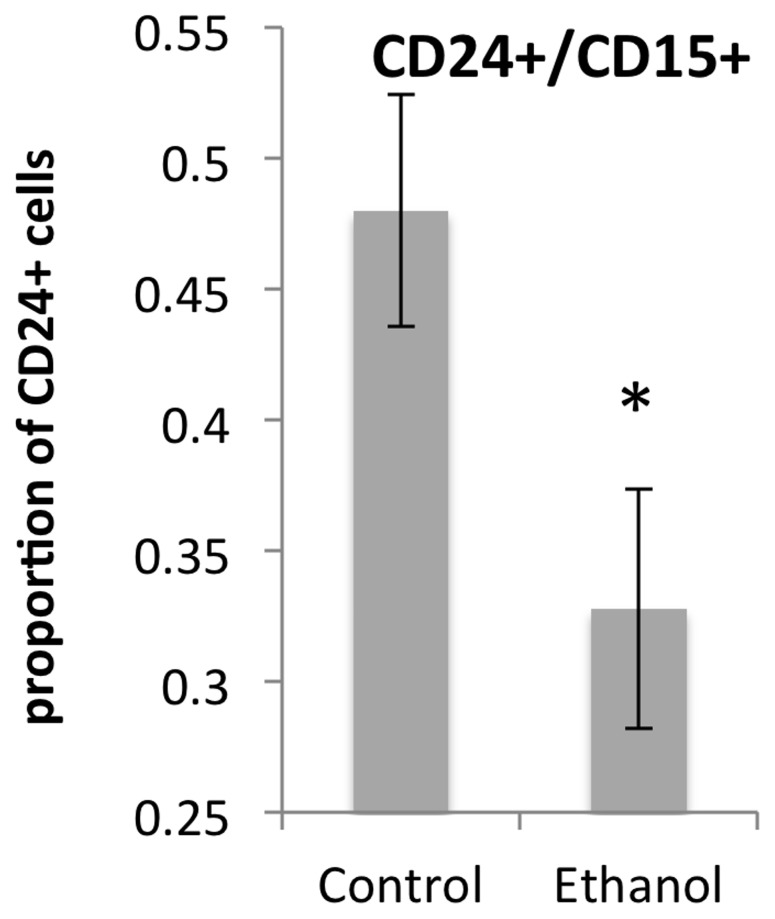
CD24^+^CD15^+^ double-positive cells are specific teratogen targets. Ethanol exposure results in a significant decrease in the proportion of CD24^+^ cells that also express CD15 (the CD24^+^CD15^+^ double-positive subpopulation). Asterisks indicate statistically significant differences relative to controls.

Neurosphere cultures were treated with ethanol or control medium for a period of 5 days, followed immediately by flow cytometric analysis of cell surface marker expression. Since ethanol exposure decreased GFAP mRNA, and since GFAP-positive NSCs with the capacity for self-renewal are also known to co-express markers for pluripotency like CD15 [52] we predicted that ethanol would decrease the expression of CD15. Ethanol exposure resulted in a specific decrease in the cell surface expression of CD15 as predicted, but additionally resulted in decreased numbers of CD24^+^ cells (**Figure 4d**). CD15 was decreased by 47%, t_(8)_  = 3.15, p<0.014, whereas CD24 was decreased by 76% t_(7)_  = 2.46, p<0.043. In contrast, CD34, CD49f and CD90 were not altered by ethanol exposure. In control neurospheres, 48±4% of CD24^+^ cells also expressed CD15 (CD24^+^CD15^+^ double-positive). The CD24^+^CD15^+^ population accounts for most of the CD15 expression in NSCs. Ethanol exposure significantly decreased the proportion of CD24^+^ cells that were CD24^+^CD15^+^ double positive (**Figure 5**, t_(8)_ = 2.39, p<0.044), suggesting that the CD24^+^CD15^+^ subpopulation was the specific target of ethanol.

### In vivo Ethanol Exposure Decreases CD24 Expression

**Figure 6:**
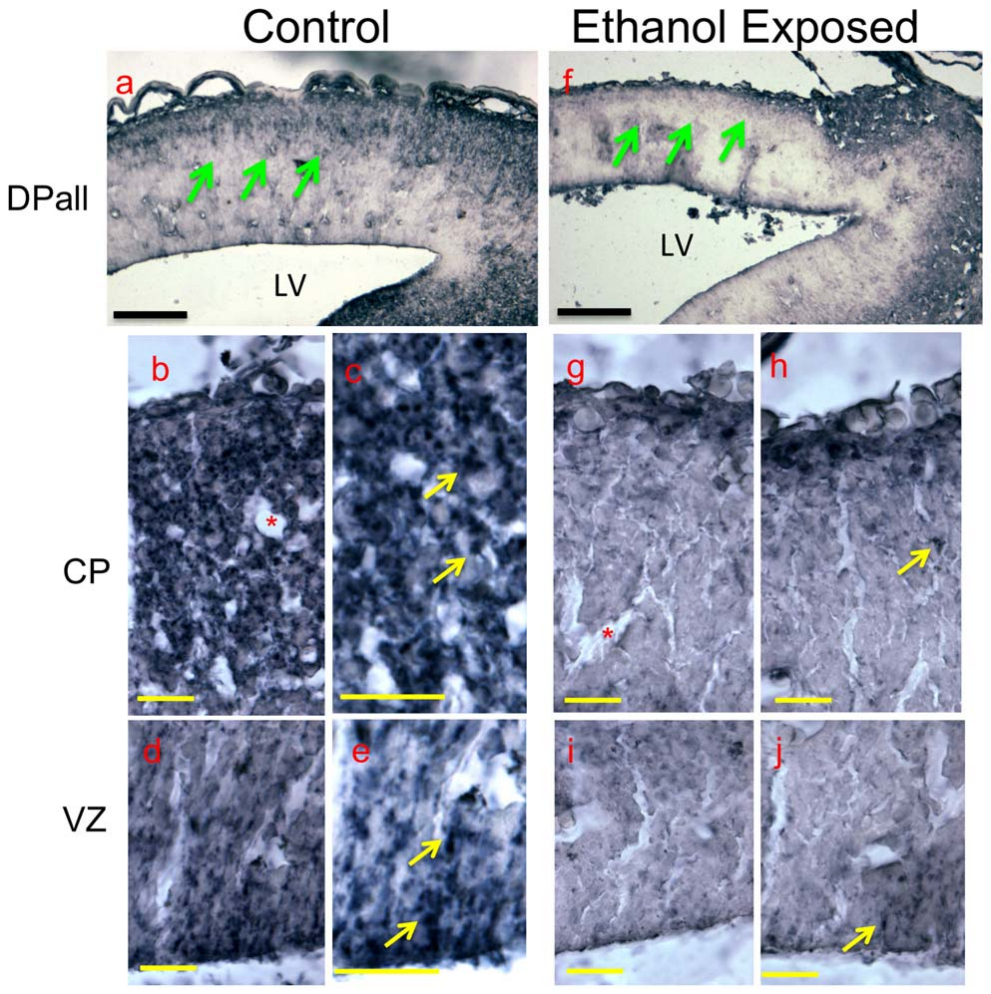
Telencephalic CD24 expression following in utero ethanol exposure. Photomicrographs showing the immuno-histochemical localization of anti-CD24 antibody immunoreactivity in tissue sections obtained from control and ethanol-exposed fetal brains. Brain sections from fetuses obtained from timed pregnant mice exposed to ethanol by intragastric gavage exhibited loss of forebrain CD24 expression compared to controls (**f** vs. **a**, green arrows). (**b–e**) Higher magnification micrographs of the control dorsal pallium/isocortex (**b,c**) and ventricular zone (**d,e)** show that CD24-immunoreactivity is localized to distinct peri-cellular puncta (yellow arrows), as expected for a cell adhesion molecule. Corresponding sections from ethanol exposed fetuses (**g–j** respectively), show loss of CD24 immunoreactivity in ethanol exposed fetuses. Asterisks show lumens of blood vessels. Scale bars, **a**,**f**, 500 µm; **b**–**e**,**g**–**j**, 25 µm. Abbreviations, DPall, dorsal Pallium; LV, lateral ventricle; CP, Cortical Plate; VZ, Ventricular zone.

Since ethanol exposure decreased CD24 expression in neurosphere cultures, we tested the hypothesis that *in vivo* exposure to multiple binge episodes of ethanol would also result in decreased CD24 expression. Timed-pregnant mice (four in each condition) were exposed to ethanol or water by intra-gastric gavage twice daily between GD12.5 and GD14.5, and euthanized at GD15. One fetus from each dam was assessed for the expression of CD24 immuno-reactivity, using the same antibody that was used for flow cytometry. In control GD15 fetal mice, patterns of CD24 immuno-labeling were similar to the mRNA expression pattern outlined in the Allen brain atlas of the GD15.5 mouse brain (http://developingmouse.brain-map.org/data/Cd24a/100046636.html, nomenclature is adapted from the Allen Atlas). Strong CD24 immunoreactivity was associated with a variety of brain regions including the ventral pallium, septopallidal and septostriatal areas, mesencephalic tectum, ponto-meduallary hindbrain, and spinal cord (data not shown). CD24-immuno-labeling was also observed in the dorsal telencephalic vesicle (the region of the dorsal pallium/isocortex, DPall), and immuno-positivity within this region was localized mainly to superficial cortical lamina (**Figure 6a** **, arrows and 6b**), populated by newly migrating and differentiating neurons. Higher magnification images show that CD24-immunoreactivity is localized to discrete puncta (**Figure 6** **,c and e**) rather than to the cellular cytoplasm, consistent with the role of CD24 as a cell adhesion molecule [54]. CD24^+^ labeling was also observed at the apical ventricular zone (**Figure 6d**), associated with linearly arranged puncta (**Figure 6e**). Ethanol exposure resulted in decreased CD24 immuno-reactivity throughout the anterior portion of the brain including the telencephalic vesicles (**Figure 6f–j** *vs.* **6a–e**). Loss of labeling was observed in both the cortical plate (CP, **Figure 6g,h**) and the ventricular zone (VZ, **Figure 6i,j**). *In utero* ethanol exposure also resulted in decreased thickness of the dorsal telencephalic pallium and resulted in dilated ventricles as we have recently shown [**16**].

### Ethanol Exposure Persistently Increases Cell Proliferation in both Wild-type and CD24^+^ NSCs

**Figure 7:**
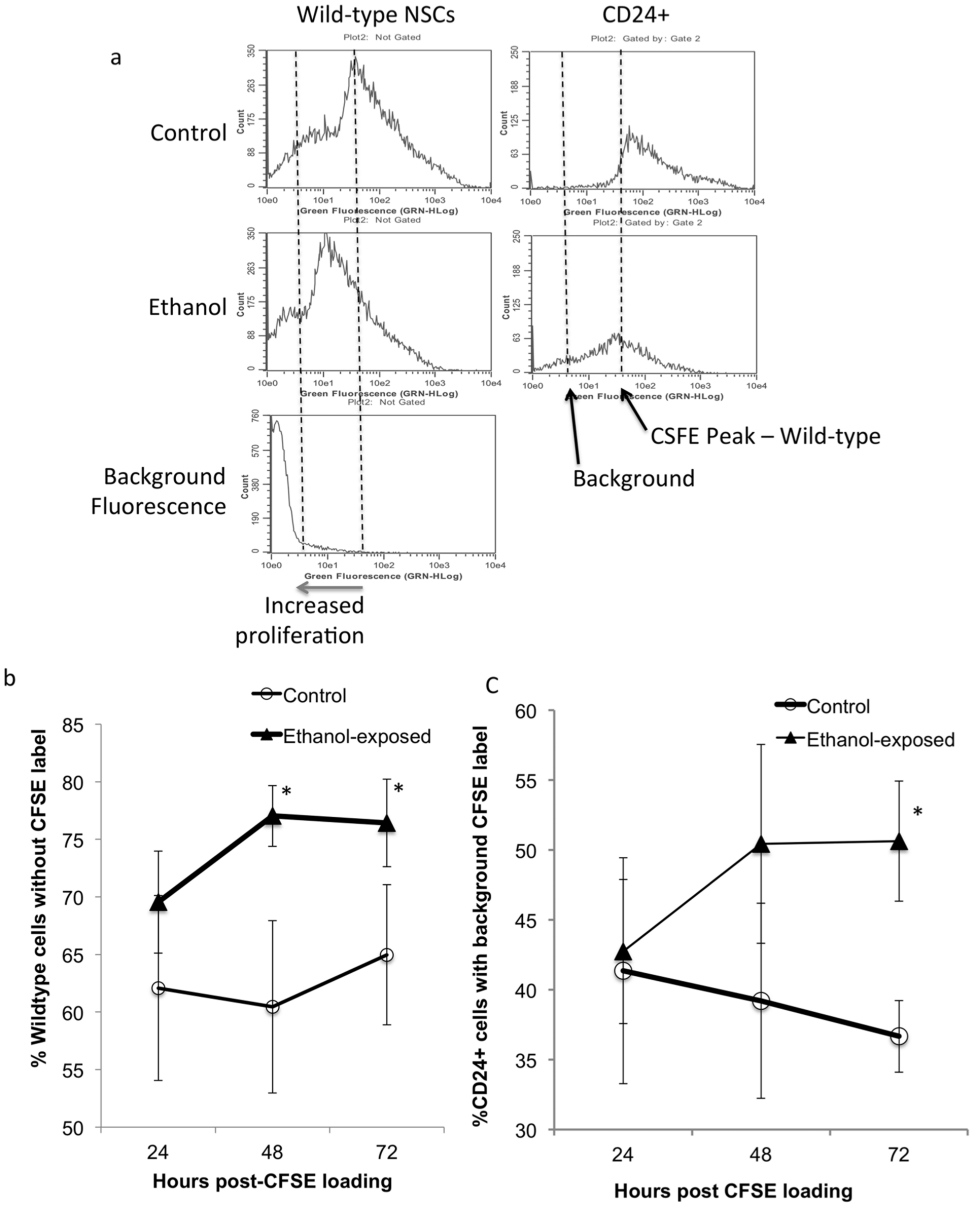
Proliferation of CD24^+^ cells. (a) Flow cytometric histograms, of CFSE-labeled cells showing proliferation following ethanol exposure. Control and ethanol treated cultures were labeled with CFSE and cultured for an additional period of up to 72 hours in the absence of ethanol. CFSE permanently labels cells and the label is diluted by successive cycles of cell proliferation, leading to a left shift in intensity. Cells were immunolabeled for CD24 and the number of cells at background staining for CSFE was quantified in all cells (left panels) and in CD24-labeled cells (right panels) as a measure of label dilution and consequently, proliferation. ‘X’-axis denotes CFSE fluorescence intensity and ‘Y’-axis denotes cell number. Histogram in bottom left panel shows background fluorescence. (**b**,**c**) Quantitative analysis shows that over a 72-hour period, both wild-type and CD24-gated ethanol-pretreated cells exhibit increased proliferation compared to controls. Asterisks indicate statistically significant differences relative to controls.

We next assessed the effects of prior ethanol exposure on the subsequent proliferation potential of the residual CD24^+^ population. Neurosphere cultures were treated with ethanol or control medium for a period of 5 days. Following cessation of alcohol exposure, cells were labeled with CFSE dye, then cultured for an additional period ranging from 24 to 72 hours. CFSE permanently labels cells, but dye fluorescence intensity is diluted by each successive cell division, so the loss of CFSE label in a cell is indicative of at least 7 previous cell division cycles [30]. At the end of each 24-hour time period, single cell suspensions were labeled with anti-CD24 antibody and then processed for flow cytometric analysis in both wild-type and CD24-gated NSC populations. Sample flow cytometric histograms show that ethanol pre-exposure induced a left shift in peak CFSE fluorescence intensity in both wild-type and CD24^+^ gated cells (**Figure 7a**) indicating an overall increase in cell proliferation. Between 48 and 72 hours following CFSE loading there was a statistically significant main effect of ethanol exposure (F_(1,16)_ = 6.85, p<0.019, **Figure 7b**) on loss of CFSE label, resulting in increased cell proliferation in wild-type NSCs. There was not a statistically significant effect of time. CD24^+^ cells were less proliferative than wild-type cells. Between 48 and 72 hours after CFSE loading, control CD24^+^ cells exhibited no further proliferative activity. However, ethanol exposed CD24^+^ cells continued to exhibit increased continued proliferation (F_(1,16)_ = 5.1, p<0.038, **Figure 7c**), which persisted at 72 hours relative to control (p<0.024). Loss of CFSE label occurs following 7 or more cell division cycles [30], and murine fetal cortical neuroepithelial cells can complete one cell division cycle in as little as 8 hours [55]. Therefore, the loss of CFSE label at 72 hours is likely to be due to at least 7–8 cell division cycles in a subpopulation of wild-type cells, and is likely explained by increased proliferation of CD24^+^ subpopulation. However, one caveat to these data is that ∼60% of ‘wild-type’ and ∼40% of CD24^+^ cells did not exhibit CFSE label at 24 hours. Since, neuroepithelial cells could have at best completed 3 cell division cycles in 24 hours, it is likely that these 40–60% of cells were unlabeled to begin with, perhaps because they were located within the core of the neurosphere, and unexposed to the dye.

**Figure 8:**
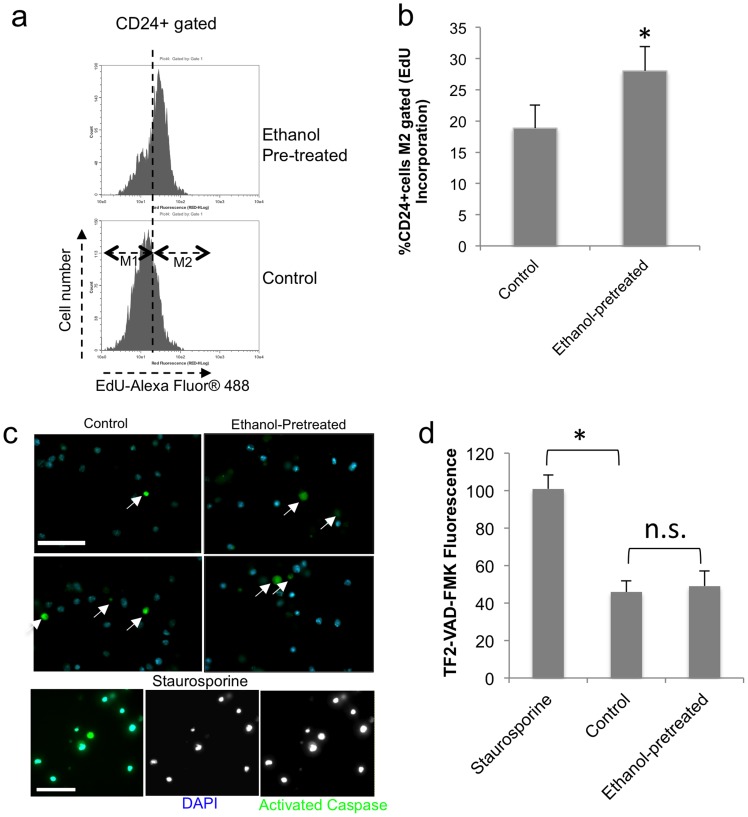
DNA synthesis and apoptosis in CD24^+^ cells. (a,b) Flow cytometric analysis of CD24-gated cells shows that ethanol pre-exposure results in significantly increased incorporation of the nucleic acid analog EdU 24 hours later: (**a**) depicts sample CD-24 gated histograms of control and ethanol pre-exposed exposed cultures, (**b**) shows quantification of percentage of EdU-positive cells (M2 gate). (**c,d**) Activated Caspase assay shows that ethanol pre-exposure does not lead to a significant change in apoptosis 24 hours later: (**c**) depicts Caspase-activated cells (green fluorescence) with DAPI (blue) as a counter-stain for nuclei. A majority of staurosporine-treated cells were apoptotic (arrows), whereas only an occasional cell was apoptotic in both the control and ethanol-pretreated conditions. (**d**) Fluorometric quantification shows that control and ethanol-pretreated cells do not exhibit a significant difference in caspase activity, and that both conditions exhibit less apoptosis than staurosporine treatment. Asterisk indicates statistical significance, n.s., not significant.

To validate the above observations, specifically in the CD24^+^ cell population, a new set of neurosphere cultures, were pre-exposed to ethanol or control medium for 5 days. Twenty-four hours following ethanol withdrawal, cultures were labeled with the nucleotide analog, EdU, for 30 minutes. Cells were fixed and labeled with the PE-conjugated anti-CD24 antibody, and the incorporated EdU was labeled with Alexa Fluor® 488 azide. Flow cytometric analysis of CD24^+^ gated cells showed significantly increased incorporation of EdU (paired t-test, t_(2) = _−5.758, p<0.029) indicating increase proliferation (**Figure 8a,b**). The EdU incorporation assay detected earlier evidence that ethanol pre-exposure resulted in increased cell proliferation in the CD24^+^ population, compared to the CFSE-loss assay. Moreover, unlike Staurosporine which induced robust apoptosis, ethanol-pretreatment did not lead to a significant alteration in apoptosis compared to control neurosphere cultures (**Figure 8c,d**, p<0.76 for the ethanol *vs.* control comparison, compared to p<0.001 for the staurosporine *vs.* control comparison) consistent with previously published data [17], [23], [24].

### CD24^+^ and CD24^depleted^ Progenitors Exhibit Divergent Renewal and Differentiation Capacity and Exhibit Differential Response to Ethanol Exposure

**Figure 9:**
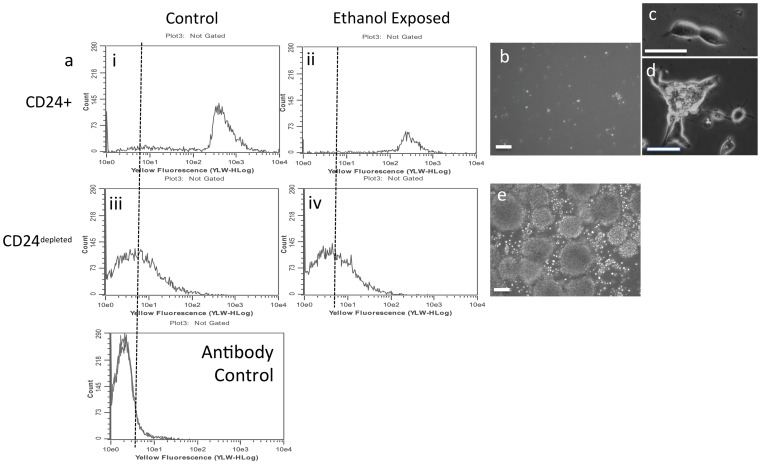
Characterization of immuno-magnetically selected CD24^+^ and CD24^depleted^ populations. (a) Flow cytometric frequency histogram of CD24^+^ (top panel) and CD24^depleted^ (middle panel) cells immuno-magnetically selected from control and ethanol-exposed cultures. ‘X’-axis shows CD24 fluorescence intensity and ‘Y’-axis shows cell number. Bottom panel shows background labeling with isotype-specific control antibody. (**b–e**) Photomicrographs of control CD24^+^ and CD24^depleted^ cells maintained for seven days in mitogenic medium (**b**,**c**) show that CD24^+^ cells do not form neurospheres in culture (**b**) but do undergo mitosis (**c**) and form small adherent cell colonies (**d**), whereas CD24^depleted^ cells form large neurospheres (**e**). Scale bar, **b**,**e**, 100 µm; **c**,**d**, 50 µm.

Wild-type control and ethanol-treated neurosphere cultures were separated by immuno-magnetic selection into CD24^+^ and CD24^depeleted^ populations. Flow cytometric analysis shows a clear separation in CD24 immuno-fluorescence intensity between CD24 immuno-selected and CD24^depleted^ neural progenitors (**Figure 9a** **i** *vs.* **iii**) indicating that these indeed constitute two distinct cell populations. Ethanol treatment also results in the recovery of fewer CD24^+^ cells (**Figure 9a** **ii** *vs.* **i**) by immuno-magnetic selection, whereas the CD24^depleted^ pool is unchanged (**Figure 9a** **iv** *vs.* **iii**), recapitulating our earlier data (**Figure 4d**).

Control immuno-magnetically selected CD24^+^ progenitors failed to regenerate neurospheres over a 7-day test period (**Figure 9b**), though they did undergo cell division to form small adherent cell clusters (**Figure 9c–d**) when maintained in mitogenic culture medium. In contrast, CD24^depleted^ cells generated abundant neurospheres from dispersed single cell suspension cultures (**Figure 9e**) during the same 7-day period. Moreover, new neurospheres could be reconstituted from single cell suspensions derived from the CD24^depeleted^ population for at least three sequential cell culture passages (data not shown). These data indicate that self-renewal capacity is retained by CD24^depleted^ but not by CD24^+^ cells.

**Figure 10:**
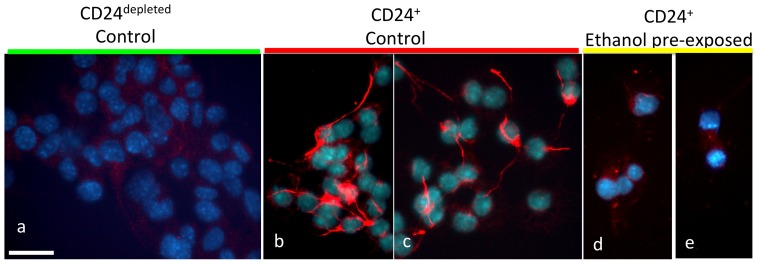
Ethanol prevents CD24^+^ cell differentiation. Photomicrographs of Map2a/b immuno-labeled control CD24^depleted^ (**a**) compared to control (**b**,**c**) and ethanol-pretreated (**d**,**e**) CD24^+^ immuno-magnetically isolated cells following mitogen-withdrawal induced differentiation. Cells are counterstained with DAPI (blue fluorescence) to visualize nuclei. Control, CD24^depleted^ cells do not undergo morphological transformation and exhibit near undetectable levels of Map2a/b immunofluorescence, showing that they are not committed to the neuronal lineage following mitogen withdrawal. Compared to control CD24^+^ cells, ethanol pre-treated CD24^+^ cells exhibited decreased neurite outgrowth and little Map2a/b immunofluorescence suggesting that they were resistant to mitogen withdrawal-induced, i.e., cell-autonomous differentiation. Scale bar, **a–e**, 25 µm.

We next asked if ethanol exposure altered subsequent differentiation potential of CD24^+^ cells. We cultured wild-type neurospheres in control medium, or in the presence of ethanol for 5 days, then used immuno-magnetic labeling to separate CD24^+^ cells from the CD24^depleted^ population. CD24^+^ and CD24^depleted^ populations were then cultured in the absence of ethanol, under mitogen-withdrawal induced differentiation conditions previously shown to promote neuronal differentiation [19] for an additional 72 hours, then assessed for the appearance of Map2a/b immunofluorescence as a marker for dendritogenesis and neuronal lineage commitment [53]. Under mitogen withdrawal-induced differentiation conditions, control CD24^depleted^ cells did not exhibit morphological transformation into neurons, nor did they express Map2a/b immuno-reactivity (**Figure 10a**); thus reinforcing our earlier data showing that the CD24^depleted^ population retained their identity as un-differentiated NSCs, even when challenged with a differentiation signal. In contrast, control CD24^+^ cells responded to mitogen withdrawal by expressing Map2a/b-immuno-positive dendrites indicating commitment to neuronal lineage (**Figure 10b**). However, pretreatment with ethanol completely inhibited the expression of Map2a/b immunoreactivity (**Figure 10c**), suggesting that ethanol exposure renders the CD24^+^ population refractory to differentiation stimuli. Moreover, ethanol pretreated CD24^+^ progenitors were unable to differentiate in the presence of 1 µM retinoic acid (data not shown) suggesting that these progenitors may have acquired a broader resistance to cue-directed differentiation signals as well.

Evidence that ethanol-pretreated CD24^+^ cells were rendered refractory to differentiation stimuli suggested that ethanol altered the intrinsic (cell-autonomous) program for lineage specification in this progenitor population. To assess this possibility, wild-type neurosphere cultures were treated with ethanol or control medium for 5 days, then subject to immuno-magnetic selection to isolate the CD24^+^ population. We examined the expression of mRNA transcripts for two neuronal lineage markers, Doublecortin and NeuroD1, and an oligodendrocyte lineage marker, Platelet Derived Growth Factor Receptor-α. Multivariate analysis (MANOVA) indicated that ethanol-treated and subsequently isolated CD24^+^ NSCs did not exhibit an alteration in the mRNA transcripts for these lineage markers (Pillai’s trace Statistic, F_(3,2)_ = 0.695, p<0.42, data not shown). Consequently, loss of lineage-specific mRNAs did not account for an alteration in the cell autonomous program for CD24^+^ differentiation.

### In Utero Adoptive Transfer Studies Show that Ethanol Pre-treatment Prevents the Survival and Integration of CD24^+^ Cells *in vivo*

**Figure 11:**
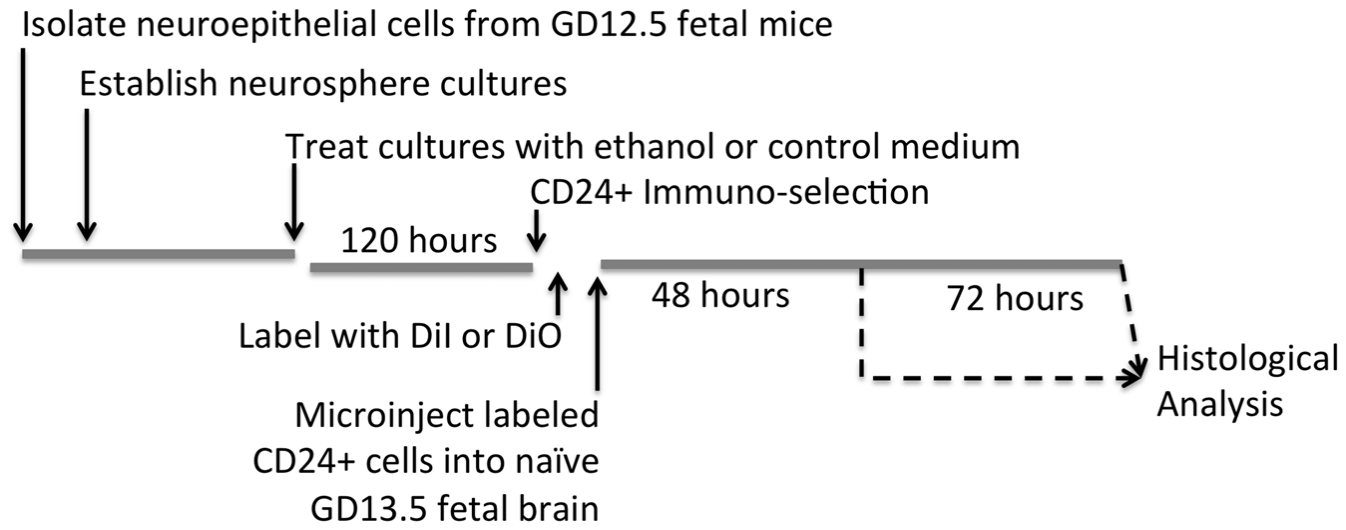
Time-line. Schematic of the time-line for the experimental protocol for ultrasound-guided, trans-uterine microinjection of immuno-magnetically isolated CD24^+^ cells.

**Figure 12:**
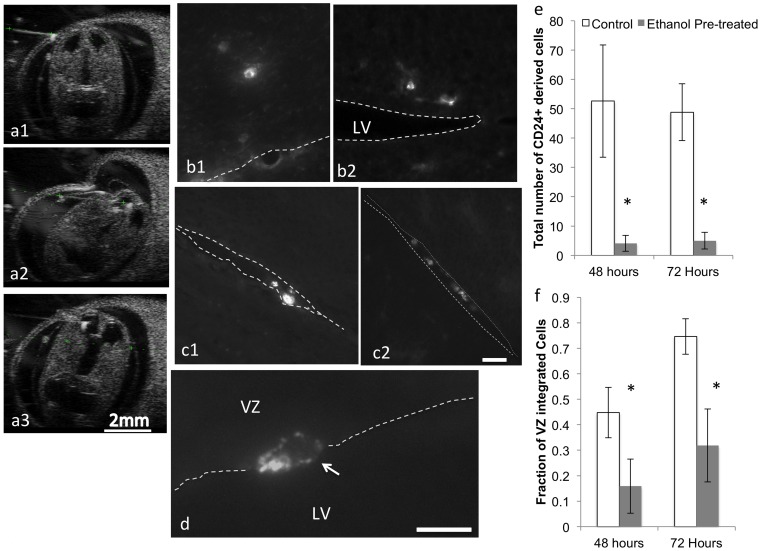
In utero orthotopic CD24^+^ cell transfer. (**a1**-**a3**) Sequential series of Ultrasound images showing trajectory of microcapillary injection of CD24^+^ cells into the lateral ventricles of GD13 mice showing pre-injection (**a1**), insertion (**a2**) and dilation of lateral ventricles (**a3**), confirming infusion of cells. Sample photomicrographs of control (**b1**–**2**) and ethanol pre-exposed (**c1–3**) CD24^+^ cells microinjected into a naïve GD13 fetal brain. Control immuno-magnetically isolated CD24^+^ cells integrate into the ventricular zone (VZ) after trans-uterine ultrasound guided microinjection, whereas ethanol pre-treated cells localize preferentially to the ventricles (**c1**–**2,** ventricles are delineated by white dashed lines) or adhere to the apical region of the VZ (**d**). (**e**,**f**) Quantitative analysis shows that total number of surviving (**e**) and integrated (**f**) CD24^+^ cells is significantly decreased in the ethanol pre-exposure condition compared to controls. Asterisks indicate statistically significant differences relative to controls. Scale bar, **b-c**, 50 µm.

To further investigate whether ethanol altered cell-autonomous programming of CD24^+^ progenitors, we performed *in utero* adoptive transfer experiments, where control and ethanol-pretreated CD24^+^ cells were re-introduced into a developmental stage-appropriate environment of the naïve fetal brain that would contain all of the extrinsic factors needed for programming the CD24^+^ cell lineage. The experimental paradigm is schematically outlined in **Figure 11**. Briefly, CD24^+^ cells were isolated from control and ethanol-pretreated neurosphere cultures by immuno-magnetic selection, tagged with a fluorescent label (DiD or DiO), and re-suspended in a mixed cell suspension containing an equal concentration of control and ethanol-pretreated CD24^+^ cells. An aliquot of these cells was aspirated into a quartz capillary micropipette and delivered into the lateral ventricle of a GD13 mouse fetus under ultrasound guidance (**Figure 12** **a1–3**). At 48 and 72 hours following fetal surgery, pregnant dams were euthanized, fetuses extracted and fixed in paraformaldehyde. Fetal brains were cryosectioned, and experimenters who were blinded to the treatment conditions, counted the number and location of labeled cells in each section.

Our data show that microinjected control CD24^+^ cells migrate from the lateral ventricles and integrate mainly into the ventricular zone (**Figure 12** **b1–2**). At GD 15 and 16, virtually no labeled CD24^+^ cells are observed in more differentiated brain regions like the fetal cortical plate. In contrast, control immuno-magnetically selected CD90^+^ cells migrate more extensively into regions like the SVZ and in the dorsal telencephalon, into the cortical plate as well (data not shown), suggesting that CD24^+^ and CD90^+^ populations constitute distinct lineages. Ethanol-pretreated CD24^+^ cells remain largely sequestered within the lateral ventricles following microinjection (**Figure 12** **c1–2;** ventricles are delineated by white dashed lines), though individual cells were often found adherent to the apical face of the lateral ventricles (**Figure 12d** **, arrow**). We observed a significant main effect of ethanol pre-treatment on the total number of CD24^+^ cells (F_(1,24)_ = 14.6, p<0.001, **Figure 12e**) indicating that fewer ethanol-pretreated CD24^+^ immuno-selected cells survived 48 and 72 hours following *in utero* adoptive transfer, compared to control CD24^+^ immuno-selected cells. There was no significant main effect of time post-injection or significant interaction effect between time and pre-exposure condition, indicating that the cell loss due to ethanol pre-exposure occurred within 48 hours following adoptive transfer. We next quantified the fraction of labeled CD24^+^ cells that integrated into the fetal VZ (**Figure 12f**). Our data show a main effect of pre-exposure condition (F_(1,24)_ = 10.88, p<0.003) as well as time following adoptive transfer (F_(1,24)_ = 4.46, p<0.045), but no interaction effect between pre-exposure and time *in utero*. These data indicate that over time, there was a significant increase in the fraction of both control and ethanol-pretreated CD24^+^ cells that integrated into the fetal VZ, however, a significantly smaller fraction of surviving ethanol-pretreated CD24+ cells integrated into the VZ at any given time.

### Following Ethanol Exposure, the CD24^depleted^ Cell Population Generates Increased Numbers of CD24^+^ cells

**Figure 13:**
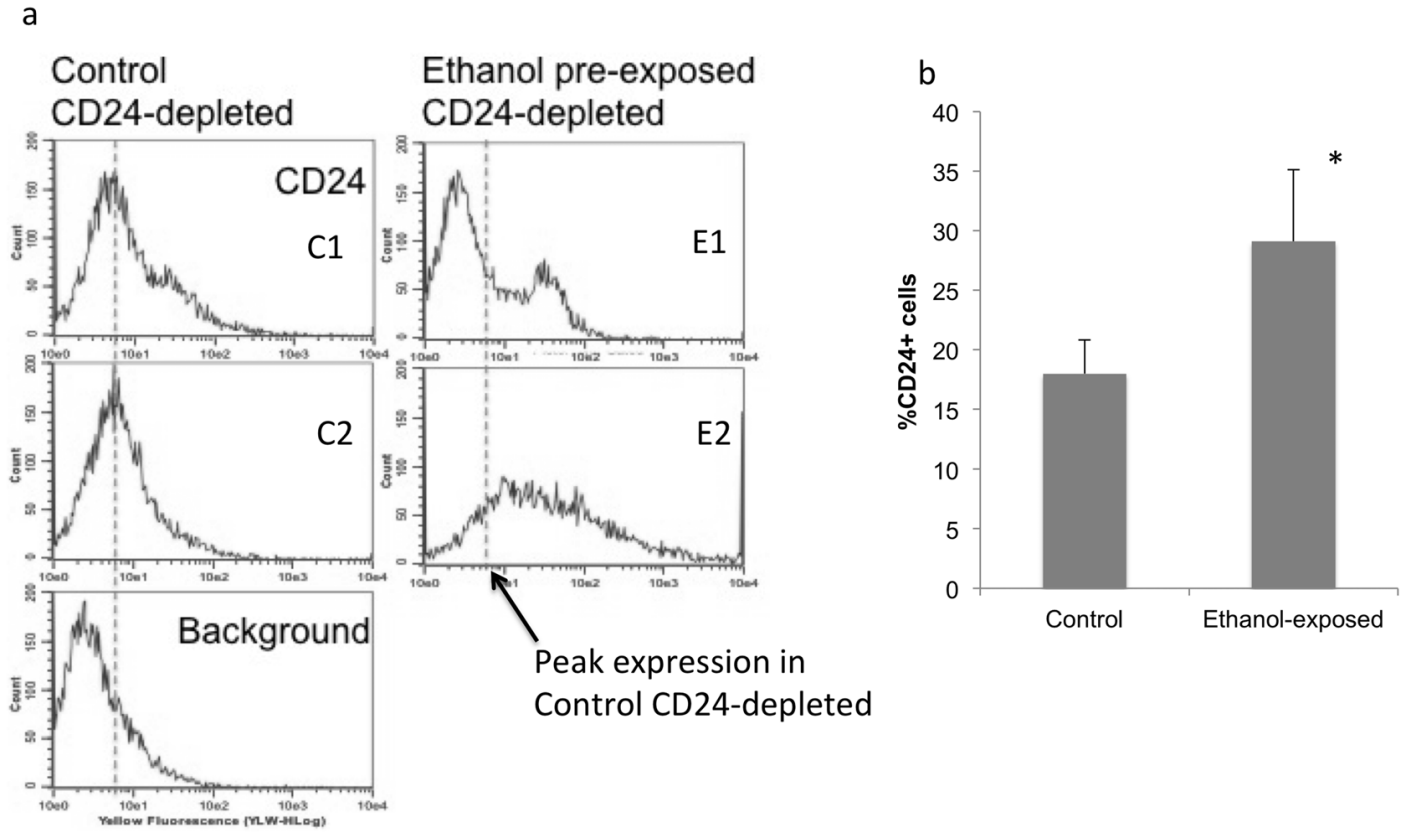
Ethanol effects on the CD24^depleted^ population. Immuno-magnetically selected CD24^depleted^ subpopulation cultured under mitogenic conditions, re-expresses CD24^+^ cells. (**a**) Flow cytometric frequency histogram showing the expression of CD24^+^ cells derived from control (left panel) and ethanol pre-treated CD24^depleted^ cells. ‘X’-axis shows CD24 immunofluorescence intensity and ‘Y’-axis indicates cell number. Bottom panel shows background immunofluorescence following labeling with an isotype-specific antibody. Vertical dashed lines show peak CD24 immunofluorescence in control cells. Sample histograms, and quantitative analysis (**b**) show variable, but significantly increased numbers of CD24^+^ cells derived from ethanol pretreated, CD24^depleted^ cells relative to controls. Asterisks indicate statistically significant differences relative to controls.

Since the CD24^depleted^ population exhibited capacity for self-renewal but not differentiation, we further examined the capacity for ethanol-pretreated, CD24^depleted^ progenitors to regenerate the CD24+ population. Neurosphere cultures were treated with ethanol or control medium for five days and the CD24^depleted^ population was immuno-magnetically selected and cultured for an additional period of 7 days in control culture medium. We observed extensive variability in the capacity of CD24^depleted^ cells to generate new CD24^+^ cells, particularly following ethanol pretreatment (e.g., **Figure 13a** **E1 vs. E2**) However, ethanol pre-treatment resulted in a modest, but statistically significant, 1.6-fold increase in the percent of CD24^+^ cells present in the cultured CD24^depleted^ population (paired t-test, t_(11)_ = -2.915, p<0.014, **Figure 13b**).

## Discussion

Teratogens are defined by their capacity to not only induce immediate changes in developing tissues (i.e., activational effects), but importantly, long-term, persistent consequences beyond the exposure period (i.e., organizational effects). For instance, ethanol exposure during pregnancy is a well-established cause of microencephaly, or decreased brain size, in humans [56]–[58], and in animal models [59]–[61]. Microencephaly could be due to immediate alcohol toxicity, resulting in the death of fetal NSCs, or due to the persistent, organizational consequence of reprogramming the developmental trajectory of NSCs. This study follows from our earlier observations that exposing NSCs to ethanol resulted in persistent effects on subsequent maturation without toxicity, i.e., without inducing necrosis or apoptosis [17], [19], [24]. These data are supported by our current observations, that ethanol pre-exposed NSCs do not undergo apoptosis. Nevertheless, when presented with a differentiation stimulus, these cells fail to express β-catenin-positive focal adhesions and Map2a/b polymerization, both essential features of dendritogenesis and neuronal lineage identity [53], [62]. However, the transformation of multipotent NSCs into neurons entails multiple steps from stem cell, to transit amplification intermediaries, to lineage commitment and diversification [25]. It is important to determine the specific identity of vulnerable NSC subpopulations, if we are to understand and perhaps mitigate teratogenesis. We therefore utilized a subpopulation analysis approach [27], [28], to identify vulnerable NSC subpopulations by their expression of unique cell surface molecular codes, and follow their developmental trajectory. Using this approach, we identified a population of cells expressing the cell surface marker, CD24, as a specific teratogen target.

We observed that ethanol exposure decreased numbers of CD24^+^ cells, a subpopulation specifically committed to neuronal differentiation [26]–[28], in dorsal pallium-derived neurosphere cultures. In contrast, CD90 which marks cells committed to both neuronal and glial lineages [63], was unaffected by ethanol exposure. Compared to the CD24^depleted^ population, which exhibits significant renewal capacity, CD24^+^ cells exhibit limited proliferation potential and lack the capacity to form new neurospheres. Moreover, following mitogen withdrawal, CD24^+^ but not CD24^depleted^ cells undergo neuronal differentiation showing that CD24^+^ cells encode a cell-autonomous differentiation program, which can be activated in the absence of additional environmental cues. *In vivo* analysis showed that CD24 was expressed throughout the developing fetal brain at GD15, with an expression pattern in control animals that matched the mRNA expression pattern published by the Allen Brain Atlas for the GD15.5 mouse brain. This suggests that CD24 broadly marks newly acquired neuronal identity throughout the brain and spinal cord. Interestingly, while CD24 is extensively expressed *ex vivo*, by cells within undifferentiated neurospheres, *in vivo*, most immunolabeling is observed outside the germinal VZ. This implies that normally, CD24 expression is accompanied by rapid delamination from the VZ and neuronal transformation, whereas in the neurosphere culture environment, continued exposure to mitogens may enforce a more immature phenotype.

Importantly, the loss of CD24 was recapitulated in an *in vivo* model of maternal ethanol exposure. Binge-like maternal exposure during the early period of fetal cortical neurogenesis resulted in decreased CD24 immunoreactivity in several brain regions including the dorsal pallium/isocortex, at GD15, the midpoint of the second trimester equivalent period. The broad loss of CD24 expression throughout the brain, appeared to spare the spinal cord, but nevertheless suggests that ethanol may promote loss of neuronal identity throughout the brain. It should be noted that, while CD24 appears to broadly mark the acquisition of neuronal identity, it is likely that its expression identifies molecularly and functionally heterogeneous cells in different brain regions that exhibit a common vulnerability to ethanol.

Ethanol also decreased the number of CD15^+^ cells in neurosphere cultures. CD15 by itself marks uncommitted multipotent NSCs [27], and therefore, its suppression would suggest a loss of early NSCs. However, the absolute numbers of CD15^+^ cells in our culture model were similar to the numbers of CD24^+^CD15^+^ double-positive cells suggesting that most CD15^+^ cells were committed to a neuronal lineage. The CD24^+^CD15^+^ subpopulation may constitute a transition stage between a pluripotent NSC and neuronal lineage commitment [27]. Moreover, ethanol exposure resulted in a decrease in the CD24^+^CD15^+^ double-positive cell population, suggesting that this intermediate stage of maturation was a specific target of ethanol. In the *in vivo* studies, we were unsuccessful in co-immunolabeling brain sections with the anti-CD15 antibody used for flow cytometric studies. Nevertheless, it would be important to identify whether this specific transitional stage within the CD24 lineage was susceptible to ethanol in the fetus as well. It is unlikely that cell death accounts for the loss of the CD24^+^CD15^+^ cell populations, since NSCs appear to be broadly resistant to cell death following ethanol exposure [17], [23], [24]. A more likely hypothesis is that ethanol alters the molecular identity of the CD24 cell lineage. Additional research is needed to identify the specific molecular characteristics and maturation potential of the CD24^+^CD15^+^ sub-population, but these data do suggest that ethanol is not globally detrimental, but rather, that specific NSC subpopulations, identified by a unique cell surface molecular code, exhibit selective vulnerability.

Ethanol exposure did not result in a complete loss of CD24^+^ cells, and therefore we examined the persistent (i.e., organizational) effects of ethanol exposure on the proliferation and differentiation potential of the residual CD24^+^ cells. Ethanol pre-exposure resulted in increased proliferation of residual CD24^+^ cells, suggesting that their capacity for differentiation may be compromised. We tested this possibility in a mitogen withdrawal paradigm of enforced differentiation. Mitogen withdrawal elicits a stereotypic neuronal differentiation response in NSCs [19], [64], implying that a ‘cell-autonomous’ differentiation program guides early lineage progression. Ethanol pre-exposure inhibited differentiation of CD24^+^ cells following mitogen withdrawal, indicating that ethanol interferes with cell-autonomous neuronal differentiation.

Ethanol pre-exposure also interfered with subsequent retinoic acid-directed differentiation of CD24^+^ cells. Thus, a history of ethanol exposure disrupts the subsequent capacity of this cell population to respond to proximal maturational cues. To further test this possibility, we conducted adoptive transfer experiments, where wild-type neurosphere cultures were treated with ethanol or control medium and CD24^+^ cells were isolated and microinjected orthotopically into the ventricles of a naïve GD13.5 fetus. Over a period of 48 hours, ethanol pre-exposed CD24^+^ cells exhibited significantly decreased survival and capacity to integrate within the ventricular and sub-ventricular zones of the naïve fetal brain as compared to control CD24^+^ cells. These data show that while ethanol is not directly toxic to NSCs, it may increase the subsequent vulnerability of NSCs to altered fetal environments. Moreover, ethanol altered the capacity of neuronal lineage directed precursors to respond appropriately to programming cues within the local environment, lending further support to a hypothesis that ethanol interferes with both intrinsic or ‘cell-autonomous’ and cue-directed differentiation programs.

Analysis of CD24 function may yield additional clues about mechanisms underlying ethanol-induced teratogenesis. CD24 is an extensively and variably glycosylated cell adhesion protein [54], that is expressed transiently at high levels in fetal and early postnatal mouse brain during the peak period of neurogenesis [65]. In the adult, CD24 expression is restricted to SVZ-derived neuroblasts, suggesting that its expression continues to be associated with neuronal lineage commitment [66]. CD24 exhibits heterophilic interactions with other neural cell adhesion molecules like L1 [54], that are independently ethanol-sensitive [67], [68], and recruits adhesion molecules to lipid rafts to promote cell motility [69]. Heterophilic CD24 interactions have been shown to prevent premature neuronal differentiation [70], and consequently, the expression of CD24 in neuronally-committed progenitors may prevent premature differentiation during their passage from the VZ to terminal destinations. Conversely, either loss of CD24 or interference with its heterophilic cell adhesion interactions may influence neural progenitor maturation and motility within the VZ, and perhaps explain the decreased integration capacity of ethanol pre-exposed CD24 cells. Interestingly, the transformation of CD24^+^ NSCs into neurons is accompanied by increased expression of miR-9 [71], a neural microRNA that, like CD24, is also suppressed by ethanol during development [18], [72], [73]. Collectively, these data indicate that CD24 plays a prominent role as a gatekeeper for neurogenesis, and may constitute part of a network of important differentiation regulatory factors that are molecular target for teratogens.

Finally, unlike CD24^+^ cells, the CD24^depleted^ cells retain NSC activity because of their capacity to reconstitute neurospheres. However, ethanol has a persistent, organizational effect on this population as well. Following ethanol exposure, the CD24^depleted^ population generated more CD24^+^ cells compared to controls. This suggests that ethanol promotes increased differentiation of resident NSCs within the CD24^depleted^ population. The mechanism underlying this differentiation response of the CD24^depleted^ population remains to be determined. However, the compensatory generation of new CD24^+^ cells is likely to deplete the pool of resident NSCs. Our data showing that ethanol decreased mRNA transcripts for musashi-1, an RNA binding protein that facilitates stem cell renewal [3], and GFAP, which is expressed by multipotent NSCs [52], further supports the hypothesized loss of NSCs. CD24^+^ cells themselves appear to act as negative regulators of NSC proliferation, since loss of CD24 has been shown to result in over-proliferation of neural progenitors [26], [43]. A potentially intriguing hypothesis that merits further investigation is that CD24^+^ precursors serve as ‘quorum sensors’ that regulate NSC maturation within neurogenic niches. In this model the utilization of stem cells is attenuated by a negative feedback loop mediated by proximal differentiated cells [74]. Teratogenic agents that deplete CD24^+^ cells may interfere with feedback repression of NSC maturation, and accelerate transformation and depletion of residual NSCs to replenish the CD24^+^ population. A loss of quorum sensing capacity would be predicted to deplete NSCs, diminishing the capacity of fetal stem cell niches to support prolonged periods of neurogenesis. This, along with a persistent diminution in cell-autonomous and cue-directed differentiation of residual CD24^+^ cells may constitute explanatory factors in microencephaly associated with fetal alcohol exposure.
